# Normotensive Glaucoma in the Fellow Eye of Patient with Unilateral Pseudoexfoliation

**DOI:** 10.3390/jcm12041593

**Published:** 2023-02-17

**Authors:** Da Young Shin, Chan Kee Park, Kyoung In Jung, Hae Young Lopilly Park, Na Young Lee

**Affiliations:** 1 Department of Ophthalmology, Eunpyeong St. Mary’s Hospital, College of Medicine, The Catholic University of Korea, Seoul 06591, Republic of Korea; 2 Department of Ophthalmology, Seoul St. Mary’s Hospital, College of Medicine, The Catholic University of Korea, Seoul 06591, Republic of Korea

**Keywords:** pseudoexfoliation, normotensive glaucoma, unilateral pseudoexfoliation, normotensive fellow eye of pseudoexfoliation

## Abstract

Purpose: To investigate the characteristics of normotensive glaucoma (NTG) in the fellow eye of patients with unilateral pseudoexfoliation syndrome (PXS). Methods: This study is a retrospective chart review. We included 313 patients with NTG. Using the 1:1 matched propensity score, only 94 well-matched patients were selected. A total of 47 NTG patients who had PXS in their contralateral eye (PXS group) and 47 NTG patients who did not (control group) were compared. The propensity score was matched based on age, mean intraocular pressure (IOP), baseline retinal nerve fiber layer (RNFL) thickness and baseline mean deviation (MD) of visual field (VF) score. The diagnosis of NTG was based on the presence of glaucomatous optic nerve head injury with VF defect, intraocular pressure less than 22 mmHg, open angles and no pseudoexfoliation material. Results: The PXS group had a higher ratio of males (34.0%) than the control group (17.0%). No significant differences were observed between the two groups in terms of CCT, axial length, untreated baseline IOP, baseline PSD of VF, systemic blood pressure and follow-up duration. The rate of RNFL thinning was significantly faster in the PXS group (−1.88 ± 2.83 μm/year) compared with the control group (−0.27 ± 5.29 μm/year) (*p* = 0.02). The progression rate of VF MD was slightly faster in PXS than in the control group, but there was no statistically significant difference (PXS group, −0.33 ± 0.90 dB/year; control group, −0.11 ± 0.84 dB/year; *p* = 0.236). Conclusions: NTG eyes with PXS showed faster RNFL thinning than did control NTG eyes.

## 1. Introduction

Pseudoexfoliation syndrome (PXS) is believed to be an important risk factor for glaucoma [1,2]. PXS is characterized by the production and progressive accumulation of abnormal fibrillar material in the tissues of the anterior segment of the eye and various other systems [2,3]. This dandruff-like material that accumulates at the pupillary margin or on the anterior lens surface is called pseudoexfoliation (PEX) material. When first detected, PXS usually only presents unilateral ocular involvement. However, the pathogenesis of PXS is systemic and bilateral, with asymmetric clinical manifestations [2,4]. It has been reported that 14% to 41% of patients with unilateral PXS develop the bilateral form [5,6,7,8]. Results of histologic studies suggest that PEX is asymmetric rather than truly monocular [4,9]. Although unilateral PXS affects only one eye, microscopic exfoliation deposits in the fellow eye were consistently detected in immunohistochemical and lectin histochemical examinations [4]. This suggests that PEX-free eyes might already have subclinical changes before PEX is observed by the ophthalmologist.

There have been several studies reporting an increased risk of developing glaucoma in the apparently normal-looking fellow eye with unilateral PXS. Puska et al. [10] reported that optic disc changes were present in both eyes of patients with unilateral PXS in normotensive conditions, even before PEX became detectable in either eye. Yarangümeli et al. [11] reported that normotensive fellow eyes of patients with unilateral pseudoexfoliative glaucoma (PXG) are at significant risk for glaucoma. These previous reports suggest that PXS may, itself, be a risk factor for glaucoma, without the contributory effect of PEX material. Impaired ocular blood flow and structural weakness have been suggested as mechanisms to exacerbate glaucoma in patients with PXS.

Therefore, PXG is considered to have a more aggressive clinical course and a worse prognosis than primary open-angle glaucoma (POAG). Previous studies have reported more-frequent visual field (VF) loss and faster progression in PXG than POAG, even in patients with similar intraocular pressure (IOP) [12,13]. If so, then the eye with NTG and PXS might differ in clinical features and prognosis from those with NTG without PXS. However, the characteristics of glaucomatous optic neuropathy with PEX-free, normotensive eyes with PXS have not been well documented.

In the present study, we investigated normotensive glaucoma (NTG) in the PEX-free fellow eyes of patients with unilateral PXS and compared those results with results from patients with NTG without PXS. The progression of glaucoma was evaluated, in terms of retinal nerve fiber layer (RNFL) thinning and changes in the VF parameters.

## 2. Methods

### 2.1. Patients

Patients’ data were collected through retrospective medical record review. This research was approved by the hospital’s Institutional Review Board and all relevant principles of the Declaration of Helsinki were followed. All patients underwent a complete ophthalmic examination, including slit lamp biomicroscopy, anterior chamber angle measurement by gonioscopy, IOP measurements using Goldmann applanation tonometry, central corneal thickness (CCT) assessment via ultrasound pachymetry (UD-800, Tomey Corporation, Nagoya, Japan), axial length measurements via ocular biometry (IOL Master; Carl Zeiss Meditec, Dublin, CA, USA), dilated stereoscopic examination of the optic disc, red-free fundus photography (Canon, Tokyo, Japan), RNFL thickness assessment using Cirrus optical coherence tomography (OCT, Carl Zeiss Meditec, Jena, Germany) and VF tests on a Humphrey field analyzer, using the Swedish Interactive Threshold Standard 24-2 algorithm (Carl Zeiss Meditec). The diagnosis of glaucoma was based on typical glaucomatous optic nerve changes (e.g., enlarged vertical cup-to-disc ratio for diffuse/focal thinning of the neuroretinal rim) and glaucomatous VF changes. Glaucomatous VFs were defined as those demonstrating two or more of the following criteria: (1) a cluster of ≥3 non-edge points in pattern deviation plots with a probability of <5% of the normal population, with one of these points having a probability of <1%, or a cluster of ≥2 non-edge points in pattern deviation plots with a probability of <1%; (2) glaucoma hemifield test results outside the normal limits; and (3) pattern standard deviation <5%. 

The mean IOP was the average of all measurements taken during the observation period. The baseline, untreated IOP was the value measured at the first visit. No patients were using anti-glaucoma eye drops at enrollment. Systolic and diastolic blood pressures (BP) of the radial artery were measured in the sitting position, using a standard automated BP cuff. Mean arterial pressure (MAP) was calculated using the following formula: MAP = Diastolic BP + 1/3 × (Systolic BP − Diastolic BP). VF and OCT examinations were performed at intervals 6 months after diagnosis of glaucoma. Only OCT images with a signal strength of 6 or higher were analyzed. 

The PEX material was confirmed by the slit-lamp biomicroscopy before or after pupillary dilatation. The “unilateral PXS” patient was defined if the PEX material was detected in only one eye. Among unilateral PXS patients, we enrolled only those who have NTG in the PEX-free fellow eye. 

### 2.2. NTG Diagnosis

The diagnosis of NTG was based on the presence of glaucomatous optic nerve head injury with glaucomatous visual field (VF) defect, IOP less than 22 mmHg during the repeated measurements taken on different days (without topical medical treatment). 

### 2.3. Control Group (NTG Eye without PXS)

The control group is patients diagnosed with NTG. They did not have PXS, and PEX was not observed in either eye. If both eyes were diagnosed with NTG, one eye was randomly selected for analysis.

### 2.4. PXS Group (PEX-Free NTG Eye)

In these patients, PEX material was detected in only one eye. If PEX-free fellow eyes have NTG, they were included in this group and analyzed.

### 2.5. Statistical Analysis

Propensity score matching was conducted to adjust for differences between the groups. Propensity scores were obtained using binary logistic regression with covariates of age, baseline RNFL thickness, baseline MD of VF score and mean IOP. Subsequently, the PXS group was 1:1 matched to the control group. Data are presented as means and standard deviations. Independent t-tests were used to compare the two groups (PXS group and control group). Chi-squared tests were used to compare categorical variables. A value of *p* < 0.05 indicated statistical significance. All statistical analyses were performed using SPSS for Windows (v. 24.0; IBM Corporation, Armonk, NY, USA).

## 3. Results

Overall, 313 NTG patients were included in this study. A total of 94 well-matched patients were selected. A total of 47 PEX-free eyes of 47 unilateral-PXS patients and 47 NTG eyes of 47 patients (not PXS) were included in the final analysis. The data from all enrolled patients are presented in Table 1. After the matching, there was no significant difference between the groups in terms of characteristics, such as age, baseline MD of VF and mean IOP. The average observation period was 6.35 ± 3.27 years.

There were no significant differences between the two groups in terms of CCT, axial length, hypertension, diabetes, dyslipidemia, migraines or blood pressure (Table 2). Baseline untreated IOP was 15.32 ± 3.22 mmHg in the PXS group and 14.23 ± 3.09 mmHg in the control group (*p* = 0.099). All subjects started anti-glaucoma eye drops after enrollment; the numbers of eye drops were 1.53 ± 0.80 in the PXS group and 1.47 ± 0.62 in the control group (*p* = 0.667). The rate of global RNFL thinning in the PXS group (−1.88 ± 2.83 μm/year) was significantly faster than in the control NTG group (−0.27 ± 5.29 μm/year; *p* = 0.037). However, the rate of MD progression showed no statistically significant difference between the two groups (*p* = 0.237). In the subsequent multivariate analysis, the rate of RNFL thinning and gender showed a difference between the two groups. Comparisons between the two groups are presented in Table 3.

## 4. Discussion

In clinical practice, many patients with PXS show only unilateral ocular involvement [14,15,16]. These PEX-free fellow eyes sometimes remain normal without glaucomatous damage. However, as the observation period increases, glaucoma quite frequently develops in PEX-free eyes.

In a previous study, Yarangümeli et al. reported that, of 60 patients with unilateral PXG, 17 patients had glaucoma in the PEX-free fellow eye (11.7%) [11]. Puska showed that, of 18 patients with unilateral PXG, 2 patients had glaucoma in the PEX-free fellow eye (11.1%), and of 35 patients with unilateral PXS, 2 patients had glaucoma in the PEX-free fellow eye (5.7%) [7]. Andrikopoulos et al. demonstrated that, of 39 patients with unilateral PXG, 11 patients had glaucoma in the PEX-free fellow eye (28.2%) [17]. They revealed that PEX-free eyes in patients with PXS have an increased risk of developing glaucoma, as do eyes with PEX. That suggests that eyes with NTG with PXS may have different clinical features than those without PXS.

Our results demonstrated that the rate of RNFL thinning was faster in the PXS group than in the control group. However, the rate of VF MD progression was not significantly different between the two groups. This is probably because the subjects had relatively early glaucoma (−6.05 ± 5.69 dB), and the follow-up period was not long (6.38 ± 3.27 years). Optic disc changes or RNFL loss appear before VF defects, so OCT is more sensitive than VFs for the detection of progression in early glaucoma [18]. Moreover, VF tests are difficult for some patients and are known to have high variability [19]. For this reason, although the progression of MD did not show statistical significance, the rate of RNFL thickness loss is sufficient to indicate the progression of glaucoma. At enrollment, there were no differences between the two groups in other risk factors for glaucoma, except for the presence of systemic PXS disease. Our results were in accordance with the previous studies that proposed that PXS itself is a risk factor for glaucoma. 

Impaired ocular blood flow and structural weakness have been suggested as the mechanisms underlying this risk. Reduced blood flow and vasculopathy are considered the result of the accumulation of PEX material within the vasculature. A study with the Heidelberg retinal flowmeter in patients with unilateral PXS revealed bilateral attenuation of microvascular flow in the optic nerve head and peripapillary retina [20]. PEX material has been detected ultrastructurally and immunohistochemically around iris blood vessels and conjunctiva in both PEX-free fellow eyes and PXG eyes [21]. An autopsy study reported early vascular change in the PEX-free eye of patients with clinically unilateral PXS, even before PEX deposits were histopathologically visible in the posterior chamber [4,9]. Due to vascular alterations, PXS has been reported to be associated with systemic disorders such as cardiovascular and cerebrovascular disease. Although the numbers of patients were relatively small in our study, there was no difference in the history of hypertension. The PXS group showed lower systolic BP and higher diastolic and mean BP than the control group, but these were not statistically significant. Large-scale, prospective, multicenter studies are needed to explore this possible mechanism.

Another hypothesis for glaucomatous ophthalmopathy in PXS is a structural weakness. An earlier study reported that optic disc changes take place in both eyes of patients with unilateral PXS [10]. PXS is a generalized basement membrane disorder, and pathologic changes have been observed in various ocular tissues. The lamina cribrosa (LC) of the eyes of patients with PXS may also be weakened by abnormal elastosis [22,23,24]. The LC is the primary site of glaucomatous optic disc damage [25]. The structural weakness of the LC caused by elastotic alterations is a risk factor for the development and progression of glaucoma, as follows. Electromicroscopic studies in cadaveric eyes with PXS showed that the stiffness of the LC was decreased [9]. Another recent study provided evidence of pseudoexfoliation-specific elastopathy by investigating the expression levels of elastic proteins, collagens and lysyl oxidases [24]. Kim et al. [26] demonstrated a thinner LC in eyes with PXG, compared with POAG. This report also demonstrated that PEX-free fellow eyes had a thinner LC, as in PXG. This suggests that apparently normal-looking fellow eyes may also demonstrate subclinical pseudoexfoliation changes and have a risk of glaucoma.

The main limitation of our study was its retrospective and hospital-based design. The sample size is small. All eyes in our study were apparently normotensive, but it cannot be ruled out that unmeasured IOP could be higher than 22 mmHg outside office hours. Our results have a high standard deviation (sometimes much bigger than the mean results). Such wide dispersion can lead to misleading inferences. Our study group showed faster RNFL thinning than did control NTG eyes. However, we did not demonstrate a statistically significant difference in progression rate in the visual field test. It is possible that the observation period of about 6 years was not long enough to show VF progression. Finally, RNFL thickness could decrease with age, even in normal individuals. The age-related RNFL thinning was reported from 0.16 to 0.37 μm/year [27,28,29,30]. Therefore, when interpreting the RNFL thinning rate in our study, the age-related effect should be considered.

Our results provide a new perspective into the management of patients with unilateral PXG. To our knowledge, this is the first study to compare the rate of progression of NTG with and without PXS. Our results show that eyes with PXS are at increased risk of glaucoma progression, even if PEX-free and with normal IOP. Our study demonstrates that examination and appropriate treatment of these eyes should be considered as well.

## Figures and Tables

**Table 1:** Baseline demographics of patients with normotensive glaucoma.

Variables	Normotensive Open Angle Glaucoma, 94 Eyes
Age, years	74.33 ± 8.30
Sex, male/female	24/70
CCT, μm	526.66 ± 41.89
Axial length, mm	23.83 ± 1.32
Hypertension, n (%)	43 (45.7%)
Diabetes, n (%)	9 (9.6%)
Dyslipidemia, n (%)	3 (3.2%)
Migraine, n (%)	6 (6.4%)
Number of glaucoma medications, n	1.50 ± 0.71
Baseline IOP, mmHg	14.78 ± 3.19
Mean IOP, mmHg	13.43 ± 2.95
Systolic blood pressure, mmHg	128.71 ± 20.21
Diastolic blood pressure, mmHg	75.37 ± 13.81
Mean arterial pressure, mmHg	92.94 ± 14.78
Baseline average RNFL thickness, μm	73.88 ± 11.16
Baseline MD, dB	−6.05 ± 5.69
Baseline PSD, dB	5.48 ± 3.99
RNFL thinning rate, μm/year	−0.95 ± 4.32
VF MD progression rate, dB/year	−0.22 ± 0.87
VF PSD progression rate, dB/year	0.14 ± 0.64
Follow-up duration, years	6.38 ± 3.27

CCT = central corneal thickness; IOP = intraocular pressure; RNFL = retinal nerve fiber layer; VF = visual field; MD = mean deviation; PSD = pattern standard deviation. Continuous data are mean ±  mean standard deviation unless otherwise indicated.

**Table 2:** Differences in demographic and clinical characteristics between patients with NTG with PXS and those without PXS.

Variable	Control Group (47 Eyes)	PXS Group (47 Eyes)	*p* Value
Age, year	73.85 ± 8.87	74.81 ± 7.75	0.579
Sex, male/female	8/39	16/31	**0.058**
CCT, μm	530.27 ± 48.31	523.05 ± 34.53	0.439
Axial length, mm	23.98 ± 1.60	23.69 ± 0.98	0.329
Hypertension, n (%)	22 (51.2%)	21 (44.7%)	0.836
Diabetes, n (%)	3 (6.4%)	6 (12.8%)	0.293
Dyslipidemia, n (%)	2 (4.3%)	1 (2.1%)	0.557
Migraine, n (%)	4 (8.5%)	2 (4.3%)	0.399
Number of glaucoma medications, n	1.47 ± 0.62	1.53 ± 0.80	0.667
Baseline IOP, mmHg	14.23 ± 3.09	15.32 ± 3.22	0.099
Mean IOP, mmHg	13.01 ± 3.14	13.76 ± 2.75	0.285
Systolic blood pressure, mmHg	129.52 ± 20.11	128.16 ± 20.53	0.798
Diastolic blood pressure, mmHg	72.28 ± 13.21	77.46 ± 13.99	0.149
Mean arterial pressure, mmHg	91.36 ± 13.59	94.00 ± 15.2	0.495
Baseline average RNFL thickness, μm	73.91 ± 12.13	73.85 ± 10.23	0.978
Baseline MD, dB	−6.04 ± 5.41	−6.05 ± 6.02	0.992
Baseline PSD, dB	5.65 ± 3.99	5.30 ± 4.02	0.671
RNFL thinning rate, μm/year	−0.27 ± 5.29	−1.88 ± 2.83	**0.037**
VF MD progression rate, dB/year	−0.11 ± 0.84	−0.33 ± 0.90	0.236
VF PSD progression rate, dB/year	0.08 ± 0.48	0.21 ± 0.77	0.337
Follow-up duration, year	6.75 ± 3.10	6.01 ± 3.42	0.277

NTG = normotensive glaucoma; PXG = pseudoexfoliative glaucoma; PXS = pseudoexfoliation syndrome; CCT = central corneal thickness; IOP = intraocular pressure; RNFL = retinal nerve fiber layer; VF = visual field; MD = mean deviation; PSD = pattern standard deviation. Statistically significant values appear in boldface.

**Table 3:** Logistic regression testing factors associated with the presence of pseudoexfoliation syndrome.

Variables	Univariant Model		Multivariant Model	
	Odds Ratio, 95% CI	*p* Value	Odds Ratio, 95% CI	*p* Value
Age, year	1.01, 0.97–1.07	0.575		
Sex, male/female	0.39, 0.15–1.05	**0.063**	0.36, 0.13–0.98	**0.045**
CCT, μm	0.99, 0.98–1.01	0.437		
Axial length, mm	0.84, 0.60–1.19	0.328		
Hypertension, n (%)	0.85, 0.39–1.86	0.692		
Diabetes, n (%)	2.15, 0.50–9.15	0.302		
Dyslipidemia, n (%)	0.49, 0.04–5.59	0.565		
Migraine, n (%)	0.48, 0.08–2.74	0.408		
No. of glaucoma medications, n	1.14, 0.64–2.00	0.664		
Baseline IOP, mmHg	1.12, 0.97–1.28	0.101		
Mean IOP, mmHg	1.08, 0.93–1.24	0.283		
Systolic blood pressure, mmHg	0.99, 0.97–1.02	0.794		
Diastolic blood pressure, mmHg	1.03, 0.98–1.07	0.152		
Mean arterial pressure, mmHg	1.01, 0.97–1.05	0.489		
Baseline average RNFL thickness, μm	0.99, 0.96–1.04	0.978		
Baseline MD, dB	1.00, 0.93–1.07	0.992		
Baseline PSD, dB	0.98, 0.88–1.08	0.667		
RNFL thinning rate, μm/year	0.62, 0.42–0.91	**0.015**	0.61, 0.41–0.90	**0.012**
VF MD progression rate, dB/year	0.99, 0.99–1.01	0.475		
VF PSD progression rate, dB/year	0.22, 0.00–127.35	0.637		
Follow-up duration, years	0.99, 0.98–1.01	0.754		

CI = confidence interval; CCT = central corneal thickness; IOP = intraocular pressure; RNFL = retinal nerve fiber layer; VF = visual field; MD = mean deviation; PSD = pattern standard deviation. Statistically significant values appear in boldface.

## Data Availability

The data presented in this study are available on request from the corresponding author. The data are not publicly available due to ethical reasons.
